# Solution-state NMR structure and biophysical characterization of zinc-substituted rubredoxin B (Rv3250c) from *Mycobacterium tuberculosis*

**DOI:** 10.1107/S1744309111008189

**Published:** 2011-08-16

**Authors:** Garry W. Buchko, Stephen N. Hewitt, Alberto J. Napuli, Wesley C. Van Voorhis, Peter J. Myler

**Affiliations:** a Seattle Structural Genomics Center for Infectious Disease, http://www.ssgcid.org, USA; b Biological Sciences Division, Pacific Northwest National Laboratory, Richland, Washington, USA; c Department of Medicine, University of Washington, Seattle, Washington, USA; d Seattle Biomedical Research Institute, Seattle, Washington, USA; e Department of Medical Education and Biomedical Informatics and Department of Global Health, University of Washington, Seattle, Washington, USA

**Keywords:** rubredoxin B, *Mycobacterium tuberculosis*, Rv3250c

## Abstract

Owing to the evolution of multi-drug-resistant and extremely drug-resistant *Mycobacterium tuberculosis* strains, there is an urgent need to develop new antituberculosis strategies to prevent TB epidemics in the industrial world. Among the potential new drug targets are two small nonheme iron-binding proteins, rubredoxin A (Rv3251c) and rubredoxin B (Rv3250c), which are believed to play a role in electron-transfer processes. Here, the solution structure and biophysical properties of one of these two proteins, rubredoxin B (*Mt*-RubB), determined in the zinc-substituted form are reported. The zinc-substituted protein was prepared by expressing *Mt*-RubB in minimal medium containing excess zinc acetate. Size-exclusion chromatography and NMR spectroscopy indicated that *Mt*-RubB was a monomer in solution. The structure (PDB entry 2kn9) was generally similar to those of other rubredoxins, containing a three-stranded anti­parallel β-sheet (β2–β1–β3) and a metal tetrahedrally coordinated to the S atoms of four cysteine residues (Cys9, Cys12, Cys42 and Cys45). The first pair of cysteine residues is at the C-terminal end of the first β-­strand and the second pair of cysteine residues is towards the C-terminal end of the loop between β2 and β3. The structure shows the metal buried deeply within the protein, an observation that is supported by the inability to remove the metal with excess EDTA at room temperature. Circular dichroism spectroscopy shows that this stability extends to high temperature, with essentially no change being observed in the CD spectrum of *Mt*-RubB upon heating to 353 K.

## Abstract 2

One third of the world’s human population is infected with *M. tuberculosis*, the etiological agent responsible for tuberculosis (TB). Here, the solution structure of the small iron-binding protein from this organism, rubredoxin B (Rv3250c), is reported in the zinc-substituted form.

## 1. Introduction   

In 2008, approximately 1.6 million people died from the infectious disease tuberculosis (TB) and another ten million people became infected (World Health Organization, 2009 ▶). The etiological agent responsible is *Mycobacterium tuberculosis*, a Gram-positive tubercle bacillus that is spread largely *via* the inhalation of droplet *M. tubercuosis* nuclei expelled from coughing infected individuals (Kaplan *et al.*, 2003 ▶). Approximately one-third of the world’s human population are infected with TB (Enarson, 2003 ▶), although most cases are in 22 ‘high-burden’ nations where the disease is endemic (Russell *et al.*, 2010 ▶). In the Western world effective public health care systems keep TB under control. Unfortunately, such protection may be fragile (Russell *et al.*, 2010 ▶) owing to the evolution of multi-drug-resistant and extremely drug-resistant *M. tuberculosis* strains along with the emergence of the human immunodeficiency virus 1 pandemic (Mitchison, 2005 ▶; Basu *et al.*, 2009 ▶). Consequently, it is of prime importance to develop a new generation of intervention strategies to treat and control TB (Palomino *et al.*, 2009 ▶; Myler *et al.*, 2009 ▶).

The current conventional TB treatment strategy employs multiple, not single, drug regimens [*e.g.* directly observed treatment and short-course (DOTS) drug therapy; Dye & Williams, 2010 ▶]. This is because it was observed that the accumulation of spontaneous genetic mutations in *M. tuberculosis* from single-drug therapy contributed significantly to the emergence of drug-resistant *M. tuberculosis* strains (David, 1971 ▶; Mitchison, 2005 ▶). To identify new anti-TB drugs, one current tactic is to focus on better understanding the molecular biology of the *M. tuberculosis* gene products, especially with regard to the interaction of various metabolic pathways in the microenvironment in the host (Russell *et al.*, 2010 ▶). Because of their likely role in electron-transfer processes, rubredoxin proteins are potential targets to be exploited as drug targets against *M. tuberculosis*. Structural information on these rubredoxins from X-ray crystallography or nuclear magnetic resonance (NMR) spectroscopy will assist rational structure-based drug design (Van Voorhis *et al.*, 2009 ▶) targeting these proteins.

Rubredoxins are small (∼6 kDa) nonheme proteins that coordinate an Fe atom tetrahedrally between the sulfhydryl groups of four cysteine residues. In addition to iron, rubredoxin may also bind cobalt (May & Kuo, 1978 ▶), nickel (Kowal *et al.*, 1988 ▶) or zinc (Blake *et al.*, 1992 ▶; Dauter *et al.*, 1996 ▶) at the metal-binding site. The *M. tuber­culosis* genome contains two tandem rubrudoxin genes: *Rv3251c* (encoding the 55-residue rubredoxin A) and *Rv3250c* (encoding the 60-residue rubredoxin B). The gene for rubredoxin B is repressed *in vitro* under mildly acidic and hypoxic conditions that mimic the state of dormant tubercle bacilli in granulomas (Kim *et al.*, 2008 ▶) and phagocytosed mycobacteria (Fisher *et al.*, 2002 ▶). Here, we report the NMR-derived solution structure of rubredoxin B (*Mt*-RubB) in the zinc-substituted state and describe some of its biophysical properties.

## 2. Materials and methods   

### 2.1. Cloning, expression and purification   

The Mt-*RubB* gene (Rv3250c/NP_217767.1) was amplified using the genomic DNA of *M. tuberculosis* H37Rv strain and the oligo­nucleotide primers 5′-GGGTCCTGGTTCGATGGTGAACGACT­ACAAACTGTTC-3′ (forward) and 5′-CTTGTTCGTGCTGTTTATTACGAGCGAGCCACCTCCACCA-3′ (reverse) (Invitrogen, Carlsbad, California, USA). The amplified Mt-*RubB* gene was then inserted into the expression vector AVA0421 at the *Nru*I/*Pme*I restriction sites, which provided a 21-residue tag (MAHHHHHHMGTLEAQ­TQGPGS-) at the N-terminus of the expressed protein. The recombinant plasmid was transformed into *Escherichia coli* BL21 (DE3) cells (Novagen, Madison, Wisconsin, USA) using a heat-shock method. Uniformly ^15^N- and ^15^N-,^13^C-labeled *Mt*-RubB was obtained by growing the transformed cells (310 K) in minimal medium (Miller) containing ^15^NH_4_Cl (1 mg ml^−1^) and d-[^13^C_6_]glucose (2.0 mg ml^−1^) supplemented with zinc acetate (6.1 µg ml^−1^) and the antibiotics chloramphenicol (35 µg ml^−1^) and ampicillin (100 µg ml^−1^). Once the cells reached an OD_600_ of ∼0.8, the medium was cooled to 298 K and supplemented with further zinc acetate (an additional 34.2 µg ml^−1^) and protein expression was induced with isopropyl β-d-1-thio­galactopyranoside (0.026 µg ml^−1^). To prepare iron-substituted *Mt*-RubB the procedure was identical except for the omission of zinc acetate and the substitution of FeCl_3_ (50 µg ml^−1^) at the start of the growth. Approximately 5 h later the cells were harvested by mild centrifugation and frozen at 193 K. After thawing and resuspension in ∼35 ml lysis buffer (0.3 *M* NaCl, 50 m*M* sodium phosphate, 10 m*M* imidazole, pH 8.0) brought to 0.2 m*M* phenylmethylsulfonyl fluoride (PMSF), the cells were passed through a French press (SLM Instruments, Rochester, New York, USA) three times. The suspension was sonicated for 60 s and then centrifuged at 25 000*g* for 1 h in a JA-20 rotor (Beckman Instruments, Fullerton, California, USA) to remove insoluble cell debris. Following filtration through a 0.45 µm syringe filter, the supernatant was applied onto an Ni–NTA affinity column (Qiagen, Valencia, California, USA) containing ∼25 ml resin. Using gravity, the column was washed sequentially with 40 ml buffer (0.3 *M* NaCl, 50 ml sodium phosphate, pH 8.0) containing increasing concentrations of imidazole (5, 10, 20, 50 and 250 m*M*). *Mt*-RubB eluted mainly in the 250 m*M* imidazole wash. The protein was con­centrated to ∼2 ml (Amicon Centriprep-10) and loaded onto a Superdex 75 HiLoad 16/60 column (GE Healthcare, Piscataway, New Jersey, USA) at a flow rate of 1.0 ml min^−1^ to simultaneously purify the protein and exchange it into NMR buffer (100 m*M* NaCl, 20 m*M* Tris–HCl, 1.0 m*M* dithiothreitol, pH 7.1). The band containing *Mt*-RubB (retention time of 82 min) was collected and the volume was reduced (Amicon Centriprep-10) to generate NMR samples in the 1–­2 m*M* concentration range (Lowry analysis) that were judged to be >95% pure by SDS–PAGE.

### 2.2. Circular dichroism spectroscopy   

A calibrated Aviv Model 410 spectropolarimeter (Lakewood, New Jersey, USA) was used to collect circular dichroism data from an ∼0.05 m*M* *Mt*-RubB sample in NMR buffer. Steady-state wavelength spectra were recorded on the same sample in a quartz cell of 0.1 cm path length at 0.5 nm increments between 200 and 260 nm at 298 K. Steady-state wavelength spectra were recorded in duplicate with a bandwidth of 1.0 nm and a time constant of 1.0 s and the average was reported. The average spectra were processed by subtracting a blank spectrum from the protein spectrum and then automatically line-smoothing the data using the Aviv software. A thermal denaturation curve was obtained by recording the ellipticity at 226 nm in 2.0 K intervals from 283 to 353 K.

### 2.3. NMR spectroscopy   

The NMR data were recorded on 1–2 m*M* samples at 293 K using Varian 750- and 600-Inova spectrometers equipped with ^1^H/^13^C/^15^N triple-resonance probes and pulse-field gradients. The data were processed with *Felix*2007 (Felix NMR Inc., San Diego, California, USA) and analyzed with *Sparky* (v. 3.115; Goddard & Kneller, 2008 ▶). Standard 2D ^1^H–^15^N HSQC, ^1^H–^13^C HSQC, HBCBCGCDHD and HBCBCGCDCHE experiments and 3D HNCACB, CBCA(CO)NH, HNCO, HCC-TOCSY-NNH and CC-TOCSY-NNH experiments from the Varian *Protein Pack* pulse-program suite were used to assign the ^1^H, ^13^C and ^15^N chemical shifts of the backbone and side-chain resonances. Chemical shifts were referenced to DSS (DSS = 0 p.p.m.) using indirect methods (Wishart *et al.*, 1995 ▶). Distance restraints for the structure calculations were obtained from 3D ^13^C-­edited aliphatic and aromatic NOESY-HSQC experiments and an ^15^N-edited NOESY-HSQC experiment using a mixing time of 80 ms. Slowly exchanging amides were identified by lyophilizing an ^15^N-­labeled NMR sample, redissolving it in 99.8% D_2_O and immediately collecting ^1^H–^15^N HSQC spectra 10, 20 and 60 min after exchange (deuterium-exchange experiment). To test the efficiency of EDTA in removing metal bound to *Mt*-RubB, an ∼1 m*M* sample of Zn-substituted ^15^N-­labeled *Mt*-RubB was treated with a 100-fold excess of ethylene­diaminetetraacetic acid (EDTA) and an ^1^H–^15^N HSQC spectrum was collected. An overall rotational correlation time, τ_c_, was estimated for *Mt*-RubB from backbone amide ^15^N *T*_1_/*T*_1ρ_ ratios (Farrow *et al.*, 1994 ▶; Buchko *et al.*, 2008 ▶).

### 2.4. Structure calculations   

The majority of the backbone and side-chain ^1^H, ^13^C and ^15^N chemical shifts for *Mt*-RubB were assigned using established protocols (Cavanagh *et al.*, 1996 ▶) and were deposited with the Biological Magnetic Resonance Data Bank (BMRB) under accession number 16473. Structure calculations were performed iteratively using *CYANA* (v.2.1; Güntert, 2004 ▶) with the chemical shift assignments and the peak-picked data from ^13^C- and ^15^N-edited NOESY-HSQC experiments as initial inputs. 37 dihedral angle restraints for both ϕ and ψ were introduced on the basis of the elements of secondary structure identified in the early structural ensembles and *TALOS* calculations (Cornilescu *et al.*, 1999 ▶). Near the end of the iterative process 34 hydrogen-bond restraints (1.8–2.0 and 2.7–3.0 Å for the NH—O and N—O distances, respectively) and ten zinc–sulfur restraints (2.2–2.4 and 3.4–4.5 Å for the Zn—S and S—S distances, respectively) were introduced into the structure calculations on the basis of proximity in early structure calculations and the observation of slowly exchanging amides (Table 1 ▶) in the deuterium-exchange experiment. In the final set of 100 calculated structures, the 20 with the lowest target function were selected and refined with explicit water (Linge & Nilges, 1999 ▶) with *CNS* (v.1.1) using force constants of 500, 500 and 700 kcal (1 kcal = 4.186 kJ) for the NOE, hydrogen bonds and dihedral restraints, respectively. For the water-refinement calculations the upper boundary of the *CYANA* distance restraints was increased by 5% and the lower boundary was set to the van der Waals limit. This water-refined ensemble of 20 structures was deposited in the Protein Data Bank (PDB) under PDB code 2kn9. Structural quality was assessed using the *Protein Structure Validation Suite* (*PSVS*; v. 1.3; Bhattacharya *et al.*, 2007 ▶) and is included in the structure-statistics summary provided in Table 1 ▶.

Note that the amino-acid sequence of *Mt*-RubB deposited in the PDB and BMRB is numbered sequentially, Met1–Ser81, starting with the 21 non-native residues at the N-terminus. Here, the first 21 non-native residues are numbered sequentially with an asterisk (Gly1*–Ser21*) and the first native residue, Met22 in the PDB and BMRB depositions, is labeled Met1. Hence, it is necessary to add 21 to the native residues described here (any residue without an asterisk) to find the corresponding residue in the PDB and BMRB depositions.

## 3. Results and discussion   

### 3.1. Solution structure of *Mt*-RubB   

**Figure 1:**
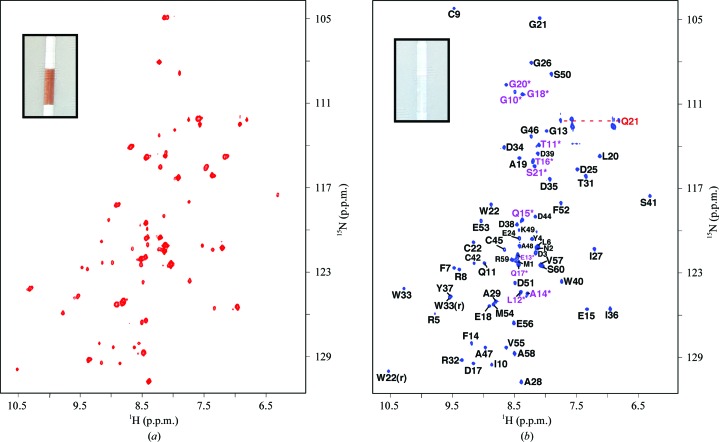
(*a*) The ^1^H–^15^N HSQC spectrum (red) of iron-associated *Mt*-RubB. (*b*) Assigned ^1^H–^15^N HSQC spectrum (blue) of zinc-associated *Mt*-RubB collected at 293 K in NMR buffer (100 m*M* NaCl, 20 m*M* Tris–HCl, 1.0 m*M* DTT, pH 7.1) at a ^1^H resonance frequency of 600 MHz. The cross-peaks for the 21-residue tag are labeled in magenta and with an asterisk. The only assigned side-chain –NH_2_ resonances are indicated by a dashed horizontal line and the exchangeable ring resonances for Trp22 and Trp33 are identified with an ‘r’ [Trp40(r) at 10.81 p.p.m. (^1^H) and 131.2 p.p.m. (^15^N) is not shown]. A photograph of the NMR tube containing the sample for the respective ^1^H–^15^N HSQC spectrum is shown as an inset.

As shown in the inset in Fig. 1 ▶(*a*), the initial sample preparation of *Mt*-RubB was reddish in color, suggesting that much of the protein had incorporated iron in the oxidized state. Such an incorporation of iron is further corroborated by the ^1^H–^15^N HSQC spectrum for this sample, as illustrated in Fig. 1 ▶(*a*). While there is good chemical shift dispersion in both the proton and nitrogen dimensions, features that are characteristic of a structured protein, the line widths of the amide cross-peaks in the spectrum are nonhomogenous, a feature that is characteristic of a bound paramagnetic species such as iron. Binding is specific and tight, as the addition of an ∼100-fold molar excess of EDTA to an NMR sample failed to change the color of the sample or the appearance of the ^1^H–^15^N HSQC spectrum (data not shown). Further indirect evidence that the *Mt*-RubB sample in Fig. 1 ▶(*a*) contained iron is that the reddish color disappeared when the protein was expressed in minimal medium lacking iron (Schweimer *et al.*, 2000 ▶) and enriched in zinc acetate, as shown in the inset in Fig. 1 ▶(*b*). More importantly, the ^1^H–^15^N HSQC spectrum of this sample (Fig. 1 ▶ *b*) still displayed good chemical shift dispersion in both the proton and nitrogen dimensions and the line widths of the amide cross-peaks were now more uniform, suggesting that the same paramagnetic species was no longer bound to *Mt*-RubB. Because NMR structure calculations are simpler if the paramagnetic effects arising from the iron can be avoided, the structure for *Mt*-RubB was determined using data from the zinc-substituted sample. Such a substitution has previously been shown to have essentially no effect on the structure relative to that of iron-substituted rubredoxin from *Clostriudium pasterurianum* (Dauter *et al.*, 1996 ▶).

**Figure 2:**
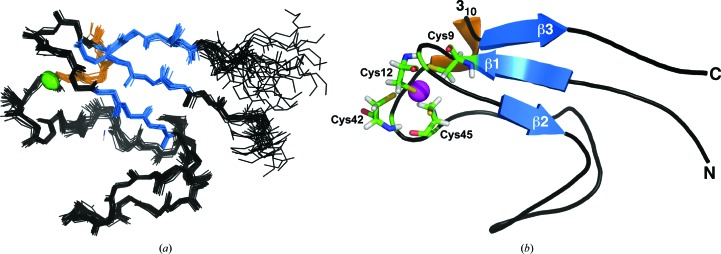
(*a*) Backbone superposition of the ensemble of 20 structures calculated for *Mt*-RubB on the structure closest to the average. The 21-residue N-terminal tag has been removed for clarity. (*b*) Cartoon representation of the structure closest to the average structure. The β-strands are colored blue, the 3_10_-helix is colored orange, the zinc ion is colored magenta and the side-chain and main-chain atoms are shown for the four cysteine residues that coordinate the metal.

In either the iron-substituted or zinc-substituted form, the elution time of *Mt*-RubB (with the 21-residue tag) on a size-exclusion column was identical (82 min) and consistent with a monomeric species. Such a conclusion was also corroborated by an estimated rotational correlation time for zinc-substituted *Mt*-RubB at 293 K (5.5 ± 0.2 ns) that was more consistent with a monomeric 9 kDa species then a 18 kDa dimer (Bhattacharjya *et al.*, 2004 ▶). As illustrated in Fig. 1 ▶(*b*), all of the amide resonances for zinc-substituted *Mt*-RubB, including residues Gly10*–Ser21* in the N-terminal tag, were unambiguously assigned in the ^1^H–^15^N HSQC spectrum. On the basis of the amide assignments and extensive assignment (94%) of the ^13^C^α^ and side-chain proton and carbon chemical shifts of residues Met1–Ser60 (BMRB ID 16473), an ensemble of structures was calculated for *Mt*-­RubB (Fig. 2 ▶ *a*) that satisfied all of the available experimental NMR data (Table 1 ▶): 892 interproton distance restraints (NOE data), 34 hydrogen-bond restraints (deuterium-exchange data), 74 dihedral angle restraints (*TALOS* calculations) and ten zinc–sulfur restraints. Each member of the final ensemble of 20 structures agreed well with the experimental data, with no upper limit violation greater than 0.05 Å and only one torsion-angle violation greater than 1°. The quality of the structure ensemble was also shown to be good using the *PSVS* validation software package (Bhattacharya *et al.*, 2007 ▶). The Ramachandran statistics for all of the residues in the ensemble were overwhelmingly in acceptable space [87.3% of the (ϕ, ψ) pairs for *Mt*-RubB were found in the most favored regions and 12.2% were within additionally allowed regions] and all of the structure-quality *Z* scores were acceptable (>−5), including the final *MolProbity* clash score of −1.26.

The final set of 20 calculated structures in the ensemble converged well, as shown mathematically by the statistics in Table 1 ▶ and visually by the superposition of the ordered residues (Asp3–Glu56) in Fig. 2 ▶(*a*). The r.m.s.d.s of the structured core of ordered residues (Asp3–Glu56) in the ensemble from the mean structure are 0.39 ± 0.07 Å for the backbone atoms (N—C^α^—C=O) and 0.76 ± 0.8 Å for all heavy atoms. The N-terminal region containing the polyhistidine tag, Met1*–Ser21*, was unstructured and disordered and, therefore, this region was omitted from the superposition shown in Fig. 2 ▶(*a*). The structure contains one three-stranded antiparallel β-sheet [β2 (Gly15–Asp17)–β1 (Leu6–Cys9)–β3 (Phe52–Val55)] and a 3_10_-helix (Ala48–Ser50) N-terminal to β3. The zinc is tetrahedrally coordinated to the S atoms of four cysteine residues (Cys9, Cys12, Cys42 and Cys45) as shown in the single structure cartoon representation in Fig. 2 ▶(*b*). Such a Cys_4_-type coordination is corroborated by the ^13^C^β^ chemical shifts for these cysteine residues. As tabulated in Table 2 ▶, the ^13^C^β^ chemical shifts for Cys9, Cys12, Cys42 and Cys45 are between 30.9 and 33.9 p.p.m., values that are in a range that is closer to the average observed for zinc-coordinated cysteine residues (30.89 ± 1.01 p.p.m.) than for reduced cysteine residues (28.92 ± 2.11 p.p.m.) (Kornhaber *et al.*, 2006 ▶).

**Figure 3:**
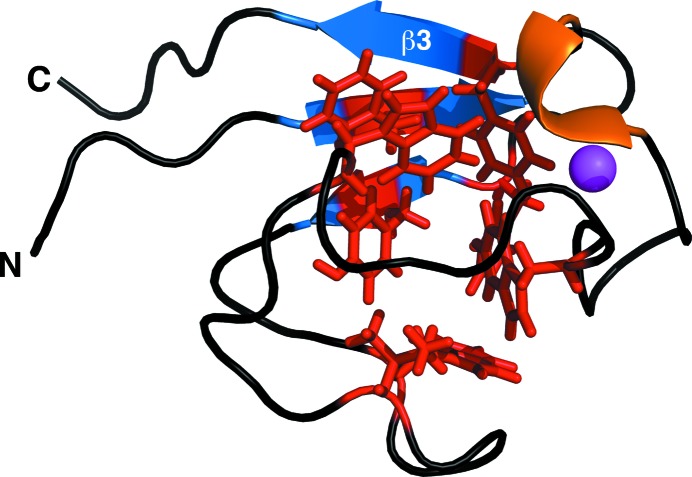
Cartoon representation of the *Mt*-RubB structure illustrating the hydrophobic core of the protein. The β-strands are colored blue, the 3_10_-helix is colored orange, the zinc ion is colored magenta and the residues of the hydrophobic core (Phe7, Phe14, Tyr16, Ile27, Trp22, Trp33, Trp40 and Phe52) are colored red.

In general, the Cys_4_-type metal coordination and three-stranded antiparallel β-sheet (β2–β1–β3) structure observed for *Mt*-RubB is similar to the structures reported for other rubredoxin proteins associated with various metals (Blake *et al.*, 1992 ▶; Sieker *et al.*, 1994 ▶; Dauter *et al.*, 1996 ▶; Schweimer *et al.*, 2000 ▶). Indeed, a search of the PDB for structures similar to *Mt*-RubB (Met1–Ser60) using the *DALI* search engine (Holm & Rosenström, 2010 ▶) generated 77 structures with *Z* scores greater than 8.4, all of which were annotated as rubredoxins. Of these 77 *DALI*-identified structures, the backbone r.m.s.d.s from the ordered region of *Mt*-RubB were 1.0 Å or less for 44 of them. Even though the *Mt*-RubB structure contains only three small β-strands and one 3_10_-helix, the small backbone r.m.s.d. of the core residues (Asp3–Glu56) in the ensemble from the mean structure (0.48 Å) suggests that the entire protein adopts a stable conformation. This has been observed in other rubredoxin structures and has been attributed to a hydrogen-bonding network between backbone amides and the S atoms of the metal-ligated cysteines and a hydrophobic core. In *Mt*-RubB there are four amide proton to cysteine sulfur bond distances of 3 Å or less (Cys12–Cys12, Gln10–Cys9, Cys45–Cys45 and Asp44–Cys42) and Fig. 3 ▶ illustrates the hydrophobic core adopted by most of the aromatic amino-acid side chains (Phe7, Phe14, Tyr16, Trp22, Trp33, Trp40 and Phe52) and the side chain of Ile27. It has been suggested that such a structural organization provides the environment for the electron-exchange reactions conducted by rubredoxins (Sieker *et al.*, 1994 ▶).

### 3.2. Thermostability of *Mt*-RubB   

**Figure 4:**
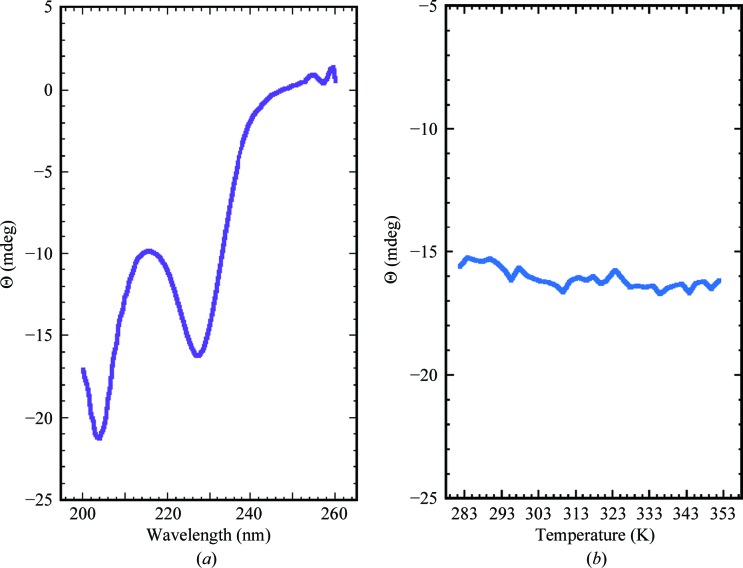
(*a*) Circular dichroism steady-state wavelength spectrum of *Mt*-RubB (∼0.05 m*M*) in NMR buffer collected at 298 K. (*b*) The CD thermal plot for *Mt*-RubB obtained by measuring the ellipticity at 226 nm in 2.0 K intervals between 283 and 353 K.

A consequence of the hydrophobic core and hydrogen-bonding network about the Cys_4_–metal center is that rubredoxins are very stable proteins (Rader, 2010 ▶). Indeed, rubredoxin from *Pyrococcus furiosus*, a hyperthermophilic archaeon, has a melting temperature of 417 K and is one of the most thermostable proteins known (LeMaster *et al.*, 2004 ▶). While the limits of the stability of *Mt*-RubB were not explored, in general, rubredoxin B from *M. tuberculosis* also appears to be a very stable protein. Fig. 4 ▶(*a*) shows a steady-state wavelength CD spectrum for *Mt*-RubB collected at 298 K. The spectrum is highlighted by a double minimum at 204 and 227 nm. The spectrum is difficult to interpret because only 14 out of 60 (23%) of the native *Mt*-­RubB residues are in canonical β-strands or 3_10_-helices and in the full-length protein 21 out of 81 (26%) residues are in the unstructured N-­terminal tag. However, the important observation is in Fig. 4 ▶(*b*), which shows the change in ellipiticity at 226 nm as a function of temperature between 283 and 353 K. There is essentially no change in the CD signal over this temperature range, indicating that *Mt*-RubB is stable to at least 353 K.

**Figure 5:**
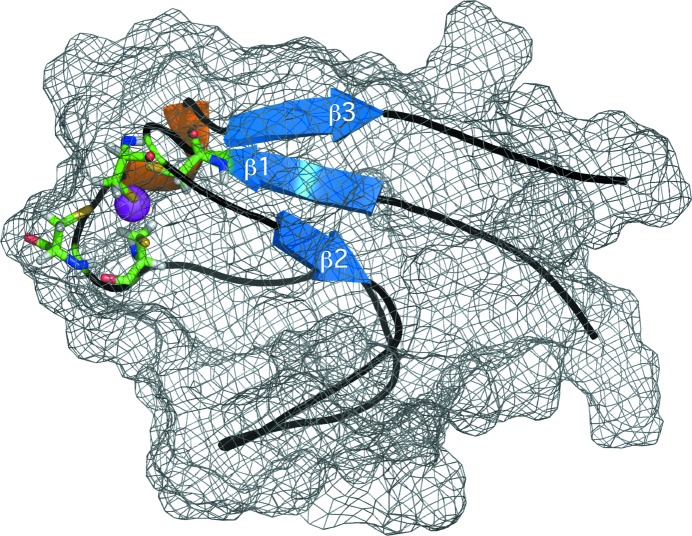
Cartoon representation of the *Mt*-RubB structure shown in Fig. 2 ▶(*b*) with the surface represented by a mesh. The β-strands are colored blue, the 3_10_-helix is colored orange, the zinc ion is colored magenta and the side-chain and main-chain atoms are shown for the four cysteine residues that coordinate the metal.

Associated with the thermostability of rubredoxins is a strong binding affinity to the metal. As mentioned earlier, the incubation of iron-substituted *Mt*-RubB (sample shown in Fig. 1 ▶ *a*) with 0.1 *M* EDTA resulted in no change in the color of the sample or the ^1^H–^15^N HSQC spectrum. Indeed, incubation of the iron-substituted *Mt*-RubB sample in the presence of 0.1 *M* EDTA for a prolonged period of time (>2 months) had no visible effect on the color of the sample or the ^1^H–^15^N HSQC spectrum (data not shown). Part of this protection from competing chelators is likely to be because the metal is buried within the protein, as shown for *Mt*-RubB in Fig. 5 ▶. Such a structural organization of the metal internally near the hydrophobic core would facilitate electron-transfer processes (Sieker *et al.*, 1994 ▶).

## 4. Conclusions   

The solution structure of zinc-substituted *Mt*-RubB, which is highlighted by a Cys_4_-type metal center and a three-stranded antiparallel β-­sheet rigidly held together by a hydrogen-bonding network and a hydrophobic core, provides the groundwork for future structure-based drug design targeting *Mt*-RubB. Circular dichroism spectroscopy shows that *Mt*-­RubB is thermostable to at least 353 K. These structural and biophysical properties of *Mt*-RubB are similar to those observed for other rubredoxins and may be universal features that are critical for the electron-transport functions of rubredoxins. When the *in vivo* electron-transfer partners for *Mt*-RubB are identified, the structure and biophysical properties presented here will assist in the molecular understanding of the protein’s biological function with its electron-transfer partner and potentially speed up the conception and development of new and improved chemotherapeutic agents to treat and control the spread of tuberculosis.

## Supplementary Material

PDB reference: rubredoxin B, 2kn9

## Figures and Tables

**Table 1:** Summary of the structural statistics for *Mt*-RubB All statistics are for the 20-structure ensemble deposited in the PDB (entry 2kn9) using the residues containing the central core (Asp3Glu56).

Restraints for structure calculations	
Total NOEs	892
Intraresidue NOEs	204
Sequential (*i*,*i* + 1) NOEs	245
Medium-range (*i*,*i* + *j*; 1 *j* 4) NOEs	157
Long-range (*i*,*i* + *j*;*j*> 4) NOEs	286
-angle restraints	37
-angle restraints	37
Hydrogen-bond restraints	34
Zincsulfur restraints	10
Structure calculations	
No. of structures calculated	100
No. of structures used in ensemble	20
Structures with restraint violations	
Distance restraint violations 0.05	0
Dihedral restraint violations 1	1
R.m.s.d. from mean ()	
Backbone NCCO atoms	0.39 0.07
All heavy atoms	0.76 0.08
All atoms	0.88 0.08
Ramachandran plot summary using *PROCHECK* (%)	
Most favored regions	87.3
Additionally allowed regions	12.2
Generously favored regions	0.4
Disallowed regions	0.0
Global quality scores†	
*PROCHECK* (all)	2.42 (0.61)
*PROCHECK* (, )	2.08 (0.41)
*MolProbity* clash score	1.26 (16.25)

† *Z* scores; values in parentheses are raw values.

**Table 2:** Comparison of the ^13^C and ^13^C chemical shifts of the cysteine residues in *Mt*-RubB with average values for oxidized, reduced and Zn-coordinated cysteine residues Average values are from Kornhaber *et al.* (2006 ▶).

Thiol	^13^C chemical shift	^13^C chemical shift
Average oxidized	57.57 2.46	41.17 3.93
Average reduced	59.25 3.06	28.92 2.11
Zinc-coordinated	59.27 2.12	30.89 1.01
Cys9	59.8 0.2	30.9 0.2
Cys12	58.8 0.2	33.9 0.2
Cys42	57.1 0.2	31.1 0.2
Cys45	59.0 0.2	33.3 0.2
